# Radio Signal Modulation Recognition Method Based on Hybrid Feature and Ensemble Learning: For Radar and Jamming Signals

**DOI:** 10.3390/s24154804

**Published:** 2024-07-24

**Authors:** Yu Zhou, Ronggang Cao, Anqi Zhang, Ping Li

**Affiliations:** 1 School of Electrical and Mechanical, Beijing Institute of Technology, Beijing 100081, China; 2 Science and Technology on Electromechanical Dynamic Control Laboratory, Beijing Institute of Technology, Beijing 100081, China; 3 Tangshan Research Institute, Beijing Institute of Technology, Tangshan 063611, China

**Keywords:** radar signal recognition, active jamming, signal classification, ensemble learning, stacking, feature engineering, random forests, K-nearest neighbor, naive Bayes, logistic regression

## Abstract

The detection performance of radar is significantly impaired by active jamming and mutual interference from other radars. This paper proposes a radio signal modulation recognition method to accurately recognize these signals, which helps in the jamming cancellation decisions. Based on the ensemble learning stacking algorithm improved by meta-feature enhancement, the proposed method adopts random forests, K-nearest neighbors, and Gaussian naive Bayes as the base-learners, with logistic regression serving as the meta-learner. It takes the multi-domain features of signals as input, which include time-domain features including fuzzy entropy, slope entropy, and Hjorth parameters; frequency-domain features, including spectral entropy; and fractal-domain features, including fractal dimension. The simulation experiment, including seven common signal types of radar and active jamming, was performed for the effectiveness validation and performance evaluation. Results proved the proposed method’s performance superiority to other classification methods, as well as its ability to meet the requirements of low signal-to-noise ratio and few-shot learning.

## 1. Introduction

Radar is a technology that utilizes electromagnetic waves to accomplish object detection and measurement of position and velocity. It is widely used in aviation, military, weather observation, environmental monitoring, geoscience, and automatic driving [1,2,3,4,5,6]. With the expansion of application scenarios and the increase in various wireless communication devices, the electromagnetic environment confronted by radar is becoming increasingly complex. In the complex electromagnetic environment, radar reconnaissance faces severe challenges. The presence of high-intensity background noise reduces the signal-to-noise ratio (SNR) of radar received signals, making it challenging for radars to distinguish targets and extract target parameters. Additionally, there may be mutual interference between different radars, regardless of whether their signal systems (such as analog modulation signal and digital modulation signal) and modulation types (such as amplitude modulation, frequency modulation, phase modulation, frequency shift keying, and phase shift keying) are the same. For instance, the use of continuous wave radar, pulse radar, and digital modulated wireless communication devices may cause confusion between real targets and false targets. In the field of electronic countermeasure (ECM), specialized jamming devices targeting radars launch active jamming, posing an all-around threat to radar. For instance, noise-modulated jamming signals can drown radar signals at specific frequencies, thereby reducing the SNR of the target echo in the radar received signal, ultimately reducing the probability of target detection and even reducing the range accuracy [7].

In order to identify the target echo signal and filter out interference from other radiation sources, it is essential to distinguish the components within the radar’s received signal. Upon confirming the existence of active jamming or mutual interference, the radar will adopt the corresponding strategy to reduce the impact. Therefore, modulation recognition for the radar received signal is of particular importance, as incorrect recognition results will lead to incorrect anti-jamming measures. This paper is concerned with the study of a modulation recognition method for potential active jamming or mutual interference signals in radar received signals.

The advancement of technology has facilitated numerous research achievements in the field of radar anti-jamming. Based on the Gaussian difference pyramid of radar signal time-frequency images, reference [8] proposes the integration of position and scale features derived from the scale invariant feature transform (SIFT) with a support vector machine (SVM) classifier to facilitate the recognition and classification of radar signals. In the context of automatic target recognition in SAR, reference [9] proposes the utilization of the dual-tree complex wavelet transform (DT-CWT) in combination with non-Gaussian statistical modeling, with the objective of overcoming the limitations of SIFT and obtaining Weibull or gamma statistical parameters as feature vectors. The combination of these features with SVM enables the method to achieve signal classification. However, the aforementioned approaches rely on image morphology features for classification, which results in limited accuracy. Moreover, the accuracy is significantly influenced by the reduction in the SNR.

Reference [10] proposes using the information entropy and singular spectrum entropy of time-domain signals as the basis for the classification task of linear frequency modulation (LFM) signals and amplitude-modulated frequency sweep jamming signals, implementing binary classification with SVM. Reference [11] proposes using the information entropy and exponential entropy of frequency-domain signals as the basis for classification, employing the fuzzy C-means method (FCM) to implement the classification task of pulse Doppler signals and jamming signals modulated by noise. These features and algorithms are relatively simple, making it challenging to identify a wider range of signal types. Moreover, feature representation and signal classification implemented solely in a single feature domain are susceptible to noise jamming, leading to performance degradation. Additionally, some researchers explored the use of higher-order moments, higher-order statistics, and higher-order cumulants of signals as features for describing different communication signal types, employing machine learning classification algorithms for automatic signal type recognition [12,13].

The advent of deep learning has led to the development of techniques based on deep neural networks that have demonstrated remarkable performance in the field of radar signal recognition. In reference [14], a robust multiscale atrous pyramid network (MAPNet) is proposed, which is capable of capturing finer-grained multiscale information in the time-frequency distribution images of radar signals. While achieving high recognition accuracy based on time-frequency distribution images and deep convolutional neural networks (DCNN), it has a high requirement for SNR. When the SNR is low, the target signal components can be overwhelmed by noise in the time-frequency distribution, leading to a significant decrease in recognition accuracy [15,16,17]. To mitigate the impact of a low SNR on recognition performance, several studies have focused on designing suppression measures specifically for noise. In reference [18], a DCNN-based noise estimation network is proposed to adaptively estimate noise intensity. This is achieved by incorporating multiscale convolution and self-attention mechanisms, which reduce the influence of noise on the target detection process in radar signals, thereby enhancing detection performance. In reference [19], a radar signal detection and synthesis mechanism is designed to generate paired clean and noisy data. A subnetwork is employed for denoising, followed by a second-stage subnetwork for classification. In reference [20], radar signal classification is implemented based on features from the time domain, frequency domain, and correlation domain, which serve as the input for a model combining a deep convolutional neural network (DCNN), a long short-term memory (LSTM) model, and a multi-layer perceptron (MLP).

Besides the effects of high-intensity noise from the complex radio background environment, two other challenges should be considered: the first is the difficulty and high cost of obtaining real samples of radar signals and jamming signals, and the second is that the composition of the dataset needs to be adjusted at any time due to the changing application scenarios, such as adding samples of new types of signals. Consequently, the challenge lies in developing a universal approach that can train algorithm models to converge rapidly from a limited number of samples while still generalizing well [21,22]. Signal modulation recognition schemes based on DNN models typically require a large volume of data and extensive computational resources for model training. Furthermore, the frequent updates of radar data are vital for monitoring and analyzing changes in the electromagnetic environment. However, this poses demanding requirements for data collection and the synchronized updating of deep neural network models [23,24]. In reference [25], a few-shot featured signal recognition scheme for blanket jamming based on meta-learning is proposed. It reduces the reliance on training sample data by scaling down the size of the DCNN model. Additionally, it incorporates manually added features, such as Holder coefficients, to describe signal frequency characteristics as auxiliary priors. The scheme utilizes the measure of feature distance for feature-based signal classification. However, reducing the model size inevitably weakens the model’s feature extraction capability, resulting in limited performance improvement.

This paper presents a classification method based on the improved ensemble learning algorithm stacking, which has been developed to meet the requirements of radar signal modulation recognition. In particular, it aims to achieve high recognition accuracy in scenarios of a low SNR and few-shot learning. The method comprises feature engineering and signal classification. The feature engineering stage generates specific features of the signal, while the classification algorithm determines the utilization efficiency of the signal features, the actual achievable recognition accuracy, and the level of computational resource consumption.

The contributions and innovations of this paper are as follows:

1. A novel approach to extract signal features from diverse domains is proposed, including the time domain, frequency domain, and chaos-fractal domains. It helps to enhance the anti-noise ability and improve the representation ability of signal characteristics.
2. The ensemble learning algorithm stacking, used for signal modulation recognition, is structurally improved by the proposed measure, namely the meta-feature enhancement, which helps to achieve better signal classification performance.
3. This paper introduces a unique configuration for the base-learner and meta-learner of stacking, ensuring applicability to few-shot learning while maintaining high classification accuracy.

The rest of the paper is organized as follows. Section 2 details the signal modulation recognition method. Section 3 introduces the simulation experiment setup and performance evaluation method. Section 4 provides the results and corresponding analysis of the simulation experiments. Section 5 analyzes and discusses the design principles and the features of the proposed method. Section 6 concludes this paper.

## 2. Signal Modulation Recognition Method

**Figure 1:**
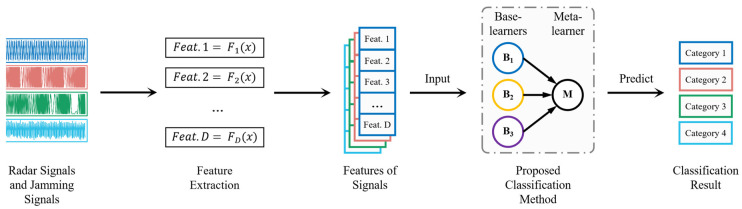
Flow chart of the proposed method.

Figure 1 illustrates the identification process of the proposed classification method based on the enhanced stacking algorithm employed for radar and jamming signals. The principal steps include feature extraction and classification. The first step is to perform the signal feature parameter extraction. The optimal feature parameters should be capable of maximally distinguishing between different types of signals. Given that the signal classification addressed in this paper must be accomplished at a low SNR and requires identification among multiple signal types, we propose the use of multiple features to describe the signal characterization, which will be detailed in Section 2.1. Finally, the signal type is predicted by the proposed method based on the aforementioned features. The proposed method will be detailed in Section 2.2.

### 2.1. Features for Classification

The similarities in characteristics among various types of signals and the challenge of the low SNR of the electromagnetic environment pose difficulties in the classification of radar and jamming signals. Describing signal characteristics solely using a specific domain or feature space is insufficient to achieve accurate recognition. Therefore, in terms of feature engineering, this paper proposes combining multiple domain features to construct a composite feature space for representing signal characteristics. Specifically, five features are introduced for the description of signal characteristics: fuzzy entropy (FE), slope entropy (SLE), spectral entropy (SPE), fractal dimension (FD), and Hjorth parameters. Fuzzy entropy and slope entropy describe the complexity and uncertainty characteristics of the signal based on the time domain. Spectral entropy describes the complexity, randomness, and uniformity characteristics of signals based on the frequency domain. The fractal dimension provides a measure of the self-similarity of a signal, which can be observed in fields of chaos and fractal geometry. Hjorth parameters offer insights into the dynamic and complex characteristics of a signal’s time-domain waveform, providing a statistical perspective. The following section provides a detailed introduction to each feature.

#### 2.1.1. Fuzzy Entropy

Fuzzy entropy (FE) is a proposed method for the analysis and identification of radar signal modulation. It is a measure of generalized information entropy used to describe the uncertainty of signal sequences. Based on the theories of information entropy and fuzzy logic, FE takes the fuzzy membership degree as a membership function to describe radar signals by the fuzzy information amount and the fuzzy measure of the information entropy. This approach allows for a comprehensive and accurate reflection of the complexity and uncertainty of the system [26]. Previously, FE has been widely applied in the analysis of electromyographic (EMG) signals, electroencephalographic (EEG) signals, electrocardiographic (ECG) signals, and mechanical vibration signals. Its use in automated applications for medical diagnosis and mechanical fault diagnosis has proven beneficial. Furthermore, FE can be applied in fuzzy control systems, fuzzy clustering analysis, fuzzy inference, and fuzzy decision-making, which makes it of significant importance. For radar signals, different modulation methods result in differences in the signal’s time-frequency distribution and uncertainty. The use of FE for the quantitative description of the complexity of different modulated radar signals could help in the analysis of signal statistics characteristics, thus facilitating the distinction of different types of signals. The calculation method of the FE is presented in Algorithm 1.
**Algorithm 1.** Calculation method for fuzzy entropy of radar signal sequences.**Define**: Signal sequence X(n),n∈[1,N]; sequence length *N*; generated sub-sequence length *m*; exponential factor *p*; similarity threshold *q***Input:** Signal sequence X(*n*)**Output:** Fuzzy entropy of X(*n*)1$X_{n}^{m}(i)=\left\{\left[x(i),x(i+1),\ldots ,x(i+m-1)\right]\right\},i\in [1,n-m+1]$ z Generate sub-sequence sets $X_{n}^{m}(i)$2$x_{0}(i)=\frac{1}{m}\sum_{k=0}^{m-1}x(i+k),i\in [1,n-m+1]$ // Obtain the mean of each sub-sequence $x_{0}(i)$3$X_{n}^{m}(i)=X_{n}^{m}(i)-x_{0}(i),\;\;\;i\in [1,n-m+1]$4$d_{ij}^{m}={max}_{k}\left|X_{n}^{m}(i+k)-X_{n}^{m}(j+k)\right|,i,j\in [1,n-m+1],i\neq j,k\in [0,m-1]$ // Obtain the maximum distance $d_{ij}^{m}$ between two sub-sequences in $X_{n}^{m}(i)$5$D_{ij}^{m}=\mu \left(d_{ij}^{m},p,q\right)=exp\left(\frac{-{\left(d_{ij}^{m}\right)}^{p}}{q}\right)$ // Calculate fuzzy membership degree $D_{ij}^{m}$6$\phi^{m}(p,q)=\frac{1}{N-m}\sum_{i=1}^{N-m}\left(\frac{1}{N-m-1}\sum_{j=1,j\neq i}^{N-m}D_{ij}^{m}\right)$7*m* = *m* + 1, repeat step 5 to obtain $\phi^{m+1}(p,q)$8${En}_{F}(m,p,q)=ln\left[\phi^{m}(p,q)\right]-ln\left[\phi^{m+1}(p,q)\right]$9Return ${En}_{F}(m,p,q)$ as the fuzzy entropy of X(n)

In Algorithm 1, for the sub-sequences $X_{n}^{m}(i+k)$ and $X_{n}^{m}(j+k)$ of a specific *i* and *j*, $d_{ij}^{m}$ is the Chebyshev distance of both, namely the maximum absolute value of the difference between the elements of sub-sequence $X_{n}^{m}(i)$. Furthermore, fuzzy membership degree $D_{ij}^{m}$ is used to measure the fuzzy similarity between $X_{n}^{m}(i+k)$ and $X_{n}^{m}(j+k)$. Based on the exponential function, $D_{ij}^{m}$ is able to take continuous values so that the FE changes continuously and smoothly.

Sample entropy and approximate entropy are calculated using the step function to measure fuzzy similarity. And there is a potential risk of abrupt changes in fuzzy similarity due to slight variations in the similarity threshold *q*. In comparison to the aforementioned limitations, the exponential function-based fuzzy membership degree allows for a more stable and robust expression of fuzzy entropy as a measure of fuzziness. Additionally, by performing mean removal on sub-sequences, fuzzy entropy avoids measurement errors caused by data fluctuations and baseline drift in signal sequences.

#### 2.1.2. Slope Entropy

Slope entropy (SLE) is a novel entropy measurement method based on symbolic patterns and slope encoding. It can be used to analyze dynamic and nonlinear characteristics and the system complexity of signal sequence. Previously, SLE has been widely applied in multivariate time series prediction, with stock price forecasting being a representative example. SLE is calculated based on the local slope of the signal. It quantifies the complexity of a signal by computing the local slopes at different time points and performing statistical analysis on these slope values. Compared to traditional entropy measurement methods such as permutation entropy and sample entropy, slope entropy (SLE) exhibits stronger robustness, lower parameter dependence, and enhanced resistance to noise [27]. Based on these characteristics, SLE is capable of capturing the dynamic variations of radar signals with non-stationary properties over different time intervals. As a result, it can provide a more comprehensive and accurate feature description.

For sub-sequence $x_{i}$ of a signal sequence X, *m* is the minimum length threshold. SLE computes the difference between the two sub-sequences $x_{i}$ and $x_{i-1}$ and then assigns the corresponding symbol. We consider the horizontal increment between $x_{i}$ and $x_{i-1}$ to always be 1, and the vertical increment are thresholded by parameter γ. The vicinity of the zero-difference region is managed by parameter δ. In this paper, γ and δ are taken as 45 degrees and 5 degrees, respectively. Furthermore, the assignment rules are as follows:

1. If $x_{i}>x_{i-1}+\gamma$, then the assigned symbol is +2;
2. If $x_{i}\leq x_{i-1}+\gamma$ and $x_{i}>x_{i-1}+\delta$, then the assigned symbol is +1;
3. For the region supposedly close to a gradient or slope 0 (equal consecutive values or ties), when $\left|x_{i}-x_{i-1}\right|\leq \delta$ (with δ close to 0), the assigned symbol is 0;
4. If $x_{i}<x_{i-1}-\delta$ and $x_{i}\geq x_{i-1}-\gamma$, then the assigned symbol is −1;
5. If $x_{i}<x_{i-1}-\gamma$, then the assigned symbol is −2.

After the symbol assignment for all required sub-sequences, SLE is defined as the sum of the Shannon entropy of all symbols, as shown in Equation (1).

(1) $$\mathrm{SLE}=-\sum_{i=1}^{n-1}p_{i,i+1}(s)\log_{2}\left[p_{i,i+1}(s)\right]$$

where p(s) represents the occurrence frequency of symbol *s*. A higher value of SLE indicates a more complex and irregular signal. For the complete calculation procedure, please refer to reference [27].

#### 2.1.3. Spectral Entropy

Spectral entropy (SPE) is an entropy measure serving for the description of the complexity and information amount based on signal spectrum. It is widely applied in image texture analysis, speech feature recognition, noise analysis, and mechanical fault diagnosis [28,29]. It quantifies the uniformity and randomness of a signal’s frequency spectrum, providing a quantitative description of the spectral characteristics of the signal.

For a signal sequence *X* = [*x*_1_, *x*_2_, …, *x*_N_], assuming Fourier transform results in Y = [*y*_1_, *y*_2_, …, *y*_N_]. The continuous frequency band $\left[f_{l},f_{h}\right]$ of the signal is randomly divided into *K* frequency intervals, and the boundaries of each frequency are determined by a vector $F_{band}=[f_{1},f_{2},...,f_{K+1}]$. Therefore, the upper and lower boundaries of the *i*-th frequency interval are determined by $f_{i}$ and $f_{i+1}$.

The power spectral intensity (PSI) of the *i*-th frequency interval is estimated as shown in Equation (2).

(2) $${\mathrm{PSI}}_{k}=\sum_{i=\mathrm{int}\left[N(f_{k}/f_{s})\right]}^{\mathrm{int}\left[N(f_{k+1}/f_{s})\right]}\left|Y_{i}\right|,k=1,2,\ldots ,K-1$$

where *f*_s_ denotes the sampling frequency and *N* denotes the length of signal sequence. Based on PSI, we define the relative intensity ratio (RIR) as shown in Equation (3).

(3) $${\mathrm{RIR}}_{j}=-\frac{{PSI}_{j}}{\sum_{k=1}^{K-1}{PSI}_{k}},j=1,2,\cdots ,K-1$$

Therefore, the definition of SPE is shown in Equation (4).

(4) $$\mathrm{SPE}=-\frac{1}{\log_{2}(K)}\sum_{i=1}^{K}{\mathrm{RIR}}_{i}\log_{2}({\mathrm{RIR}}_{i})$$

As the power spectral distribution of a signal becomes more uniform, the intensities of its frequency components become more similar. This results in higher signal complexity and a greater amount of information contained within the signal’s spectrum. Consequently, the spectral entropy value also increases. Conversely, a low spectral entropy value indicates that the energy distribution in the signal’s spectrum is more concentrated, indicating higher regularity.

#### 2.1.4. Fractal Dimension

The distribution range of complexity in radar signals varies with the modulation type. In addition to the entropy features mentioned above, we propose the use of fractal dimension (FD) to analyze the complexity characteristics of radar signals. FD measures the self-similarity of a signal sequence at different scales. It characterizes fractal patterns by quantifying the ratio of detail variations to scale variations, thereby quantifying the complexity of an object. FD was initially utilized for the quantitative description and analysis of the self-similarity of images. It later found applications in medical diagnostics, such as EEG signals and medical images [30,31,32,33]. In the case of radar, the difference in self-similarity between regularly modulated signals and blanket jamming signals with irregular noise characteristics is quite noticeable. For one-dimensional signals, we can interpret self-similarity as the degree to which the periodicity of a radar signal is disrupted by aperiodic jamming or noise. A higher FD indicates higher signal complexity, corresponding to a greater degree of disruption in the periodicity of the radar signal. On the other hand, a lower FD represents simpler patterns of signal variation, corresponding to a lower degree of disruption in the periodicity of the radar signal. In this study, the Petrosian fractal dimension (PFD) and Higuchi fractal dimension (HFD) are utilized to describe the complexity of the signals.

The PFD simplifies the calculation of fractal dimension by converting the signal into a binary representation and approximating the fractal dimension with the count of symbol changes. The definition of the PFD for a time signal sequence is shown in Equation (5).

(5) $$\mathrm{PFD}=\frac{\log_{10}N}{\log_{10}N+\log_{10}\left(\frac{N}{N+0.4\;\times \;M}\right)}$$

where *N* represents the signal sequence length and *M* denotes the number of sign changes in the signal derivative.

The calculation of the HFD begins by constructing a new sequence *X*_s_ from the signal sequence *X* = [*x*_1_, *x*_2_, …, *x_N_*], as shown in Equation (6).

(6) $$X_{s}=[x_{m},x_{m+k},x_{m+2k},\ldots ,x_{m+\mathrm{int}\left[(N-m)/k\right]k}],m=1,2,\cdots ,k$$

The length of the new sequence $X_{s}$ can be calculated using Equation (7).

(7) $$L(k)=\frac{1}{k}\sum_{m=1}^{k}\frac{\sum_{i=2}^{\mathrm{int}\left[\frac{N-m}{k}\right]}\left|x_{m+ik}-x_{m+(i-1)k}\right|(N-1)}{\mathrm{int}\left[\frac{N-m}{k}\right]k}$$

Ultimately, the least square method is used to determine the HFD using Equation (8).

(8) $$\mathrm{HFD}=\frac{\ln\left[L(k)\right]}{\ln(1/k)}$$

#### 2.1.5. Hjorth Parameters

The Hjorth parameters serve as a tool for characterizing time-domain features of signals. Their simplicity in calculation and robust results have led to widespread applications in neuroscience and mechanical sensing diagnostics. These parameters are commonly employed to analyze various signals, such as biological electrical signals and mechanical sensing signals, to reveal specific signal characteristics [34,35,36,37]. 

In this study, we explore the application of two indicators of Hjorth parameters to describe the time-domain features of radar signals, which include mobility and complexity. Hjorth mobility (HM) measures the frequency characteristics and dynamic behavior of the signal. Hjorth complexity (HC) reflects the waveform characteristics and irregularity of the signal. For a signal sequence *X*, the calculation methods for HM and HC are shown as Equations (9) and (10), where X′ and X′′ denote the first-order and second-order derivatives of signal *X*.

(9) $$\mathrm{HM}(X)=\sqrt{\frac{\mathrm{var}(X^{\prime })}{\mathrm{var}(X)}}$$

(10) $$\mathrm{HC}(X)=\frac{\mathrm{HM}(X^{\prime })}{\mathrm{HM}(X)}=\sqrt{\frac{\mathrm{var}(X)\mathrm{var}(X^{\prime \prime })}{\mathrm{var}(X^{\prime }{)}^{2}}}$$

### 2.2. Improved Classification Algorithm Based on Stacking

The task of radar signal modulation recognition is constrained by a low SNR and small-sample learning. This poses a severe challenge for the accuracy and generalization performance, which is hard for traditional machine learning methods. Therefore, we propose a more powerful algorithm based on the ensemble learning framework stacking to meet the task requirements.

#### 2.2.1. Ensemble Learning and Stacking

Ensemble learning is an important method in machine learning that aims to enhance the generalization capability and prediction performance of algorithm models by constructing multiple models and integrating their prediction results. The individual models used for combination often fail to achieve the desired optimal performance. For instance, they may exhibit low bias and high variance, resulting in insufficient accuracy in terms of prediction error performance, or they may exhibit low variance and high bias, leading to a lack of generalization ability.

Typical methods of ensemble learning include bagging, boosting, and stacking. Bagging is a technique that involves constructing multiple datasets by sampling with replacements from the original training set. These datasets are used to train multiple models, and the predictions from these models are then averaged. Bagging-based methods have smaller variance, but the generated models are similar and highly correlated. Boosting changes the weights or sample distribution of the training dataset according to certain rules, trains multiple models sequentially, and combines them through linear weighting. Boosting-based methods focus on samples with larger errors during the initial training, which can reduce bias but may lead to over-fitting [38,39]. 

Stacking trains multiple base-learners and uses their prediction results as features for the meta-learner, which is trained for the final prediction. The base-learners are used to establish hypotheses between the raw data and the class labels. Therefore, it requires the classification algorithm models to have strong information extraction capabilities and the ability to uncover underlying patterns in the input data. The meta-learner combines the hypotheses made by the base-learners based on the best fusion strategy and outputs the classification results. The meta-learner requires strong interpretability and relatively simple learners to avoid over-fitting. Therefore, commonly chosen algorithms for the meta-learner include linear regression, logistic regression, and decision trees [40,41].

From the above introduction, two major distinctions between stacking and the other two methods are evident: 

(1) Homogeneous integration and heterogeneous integration: The multiple sub-models built by bagging and boosting are based on the same algorithm, and this may amplify the shortcomings of that algorithm. In contrast, stacking allows sub-models to be different algorithms, which enables them to complement each other’s strengths, reducing prediction bias and variance. Thus, stacking possesses superior generalization capability.

(2) Decision fusion: In terms of the handling of outputs from multiple sub-models, bagging employs averaging for decision fusion, and boosting uses linear weighting, while stacking achieves decision fusion by training a meta-learner under the guidance of minimizing the loss function. This comprehensive approach effectively addresses the relationships among varying base-learners, thus finding the optimal fusion rules.

#### 2.2.2. Proposed Method

We propose an improved stacking-based classification algorithm. The training and inference process of the proposed algorithm is shown in Algorithm 2.
**Algorithm 2.** Signal modulation classification algorithm based on improved stacking.**Define**: (1) The dataset consists of a training set *T* and a validation set *V* with sample sizes *t* and *v* respectively; (2) The features proposed in Section 2.1 are defined as base-features *F* with the dimension *f*, and the class labels of the signal samples are taken as the expected outputs; (3) The number of folds for cross-validation is denoted as *K*, and the corresponding initialization index *k* is set to 1. The number of base-learner types is denoted as *N*, and the corresponding initialization index *n* is set to 1.**Input:** Base-features**Output:** Modulation type of signal**Training Phase****Training of base-learners:**1Divide the training set *T* into *K* parts equally; the *k*-th part serves as the validation set *Ta_k_* for the base-learner, containing
$\frac{t}{K}$ samples; the remaining *k*−1 parts serve as the training set *Tb_k_* for the base-learner, containing $\frac{(K\;-\;1)t}{K}$ samples2**while** (*k* < *K*) **do**3  **while** (*n* < *N*) **do**4    Initialize base-learner $B_{n}$5    $B_{\left(n,\;k\right)}=train(B_{n},\;Tb_{k})$ // Train base-learner $B_{n}$ on training set $Tb_{k}$ to obtain the corresponding pre-trained base-learner $B_{\left(n,\;k\right)}$6    $Q_{\left(n,\;k\right)}=B_{\left(n,\;k\right)}(F_{k})$ // Input base-features $F_{k}$ to pre-trained base-learner $B_{\left(n,\;k\right)}$ to obtain the corresponding output $Q_{\left(n,\;k\right)}$7**end while**8$Q_{k}=[Q_{\left(1,\;k\right)},Q_{\left(2,\;k\right)},Q_{\left(3,\;k\right)},\cdots ,Q_{\left(N,\;k\right)}]$ // Concatenate $\left[Q_{\left(1,\;k\right)},Q_{\left(2,\;k\right)},Q_{\left(3,\;k\right)},\cdots ,Q_{\left(N,\;k\right)}\right]$ along the feature axis to obtain the *k*-th fold feature $Q_{k}$9Meta-feature enhancement: $P_{k}=[Q_{k},F_{k}]$ // Concatenate $Q_{k}$ and $F_{k}$ along the feature axis to obtain the enhanced meta-feature $P_{k}$10**end while**11$P=[P_{1},P_{2},P_{3},\cdots ,P_{K}]$ // Concatenate $\left[P_{1},P_{2},P_{3},\cdots ,P_{K}\right]$ along the sample axis to obtain the meta-feature set *P*, which is based on training set *T*12Save all pre-trained base-learners $B_{\left(n,\;k\right)}$**Training of meta-learner:**13Initialize the meta-learner M14$M=train(M,\;P,\;O_{T})$ // Take the meta-feature set *P* as the input of meta-learner, take the labels of training set *T*, i.e., ground truth, as the target output $O_{T}$ for meta-learner, and then obtain pre-trained meta-learner by training15Save the pre-trained meta-learner**Validation Phase**1Load all pre-trained base-learner $B_{\left(n,\;k\right)}$ and pre-trained meta-learner M2**while** (*n* < *N*) **do**3  **while** (*k* < *K*) **do**4  $Q_{\left(n,k\right)}=B_{\left(n,k\right)}(F_{V})$ // Input the base-feature $F_{V}$ of validation set *V* into all pre-trained base-learners $B_{\left(n,k\right)}$, and then obtain output $Q_{\left(n,k\right)}$5
  **end while**
6  $Q_{n}={Voting}_{k}(Q_{\left(n,1\right)},\;Q_{\left(n,2\right)},\;Q_{\left(n,3\right)},\;\cdots ,\;Q_{\left(n,K\right)})$ // Apply the voting method to $Q_{\left(n,k\right)}$ to obtain $Q_{n}$7**end while**8Meta-feature enhancement: $P_{V}=[Q_{1},Q_{2},Q_{3},\cdots ,Q_{n},F_{V}]$ // Concatenate $\left[Q_{1},Q_{2},Q_{3},\cdots ,Q_{n}\right]$ and $F_{V}$ along the feature axis to obtain enhanced meta-feature $P_{V}$9${R=M(P}_{V})$ // Input $P_{V}$ into pre-trained meta-learner *M* to obtain prediction *R*10$E=eval(R,O_{V})$ // Evaluate the prediction performance of *R* by comparing with the target output $O_{V}$ of the validation set *V* using relevant metrics11Return the prediction *R* and evaluation results *E*

As shown in Algorithm 2, the input to the base-learners of stacking is the base-features, which are introduced in Section 2.1. The output of the base-learner is referred to as the meta-feature, which serves as the input to the meta-learner. In the original stacking algorithm, the meta-feature only contains preliminary hypotheses about the sample class based on the outputs of the base-learners. Due to their limitations, the base-learners may not be able to accurately extract the underlying patterns and effective features from the original data, resulting in hypotheses that deviate from the ground truth. Furthermore, invalid hypotheses would lead to erroneous inference by the meta-learner, ultimately producing incorrect prediction results.

In Algorithm 2, we introduce the meta-feature enhancement and incorporate it into both the training and inference stages. Meta-feature enhancement is used to enrich the amount of information contained by the meta-features, thereby improving the prediction accuracy of the meta-learner. This operation corresponds to step 9 of the training stage and step 8 of the inference stage.

**Figure 2:**
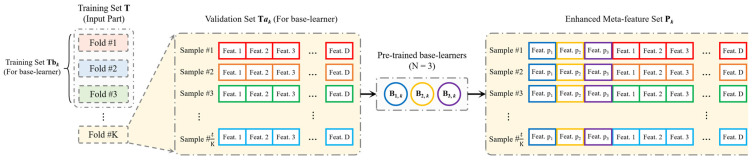
Schematic diagram of meta-feature enhancement in the training phase of the proposed algorithm.

**Figure 3:**
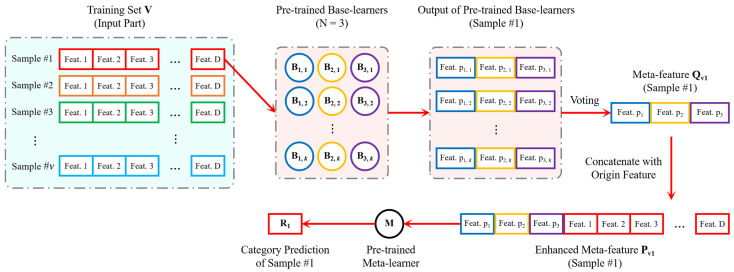
Schematic diagram of the inference flow of the proposed algorithm.

Figure 2 and Figure 3 illustrate the implementation principles of meta-feature enhancement in the training and inference stages of the proposed algorithm model, respectively. In the training stage, meta-feature enhancement is used to process the inference results from *N* base-learners, where each base-learner has one instance. In the inference stage, meta-feature enhancement is also used to process the inference results from *N* base-learners, but at this stage, each base-learner has *K* instances, corresponding to step 12 of the training phase in Algorithm 2. For a sample’s meta-feature set $Q_{\left(n,k\right)}$ with a shape of *K* × *N* from *K* base-learners’ outputs of the same class, it is first reduced to $Q_{n}$ with a shape of 1 × *N* using the voting method and then undergoes meta-feature enhancement.

#### 2.2.3. K-Fold Cross-Validation in Training Phase

As shown in Algorithm 2 and Figure 2, cross-validation is used in stacking. However, it is different from the usual cross-validation, which is embodied in the following two aspects:

(1) Different usage

In general, cross-validation is commonly used to evaluate the performance of a trained model. However, in stacking, cross-validation is employed for training the base-learners and generating the training data for the meta-learner, which are also called the meta-features. Stacking consists of several base-learners and a meta-learner, forming a two-stage model. This means that the output of the base-learners serves as the input for the meta-learner. Since the dataset does not include the meta-features used for training the meta-learner, the meta-learner needs to be trained after the base-learners have completed their training. This ensures that the meta-learner receives valid meta-features during training.

(2) Different target

In the approach of traditional K-fold cross-validation, the entire dataset is typically divided into *k* mutually exclusive folds. Then, the model is trained and validated for k rounds, with each round using one fold as the validation set while the remaining folds are used for training. This process ensures that each fold is used as the validation set exactly once across the k rounds. The final evaluation result is obtained by averaging the evaluation results from each round of validation. This approach helps provide a more reliable estimation of the model’s performance by leveraging multiple non-overlapping subsets of the data for training and validation.

In stacking, the dataset is initially split into a training set T and a validation set V, which are used for training and validating the entire stacking model, respectively. Cross-validation further divides the training set *T* into *K* subsets, where the *i*-th subset serves as the validation set for the base-learner (0<i≤K), while the remaining *K* − 1 subsets are used as the training set for the base-learner. As a result, each base-learner can be trained to generate K pre-trained models, leading to a total of N×K pre-trained models obtained by N base-learners through training.

In summary, traditional cross-validation is applied to the entire algorithm model, while cross-validation in stacking is applied to the base-learners.

#### 2.2.4. Base-Learners and Meta-Learners

In this paper, the base-learners for the proposed stacking model include three classification algorithms: random forests, K-nearest neighbors, and Gaussian naive Bayes. Moreover, logistic regression is employed as the meta-learner. A brief introduction to each algorithm and the reason why it is used are detailed as follows.

##### Random Forests

Random forests (RF) is a popular ensemble learning method based on Bagging. It is composed of multiple decision trees, and the final prediction is obtained by aggregating the individual decisions of each tree through voting [42]. RF has several advantages, such as high efficiency, strong interpretability, high parallelism, and good performance with small-scale datasets. By utilizing random sampling and random feature selection, RF effectively mitigates over-fitting, exhibits robustness against data noise, and demonstrates low variance in prediction performance. Therefore, RF exhibits excellent generalization ability. Moreover, thanks to the ability to train each tree in parallel, RF exhibits a significant speed advantage, particularly when dealing with large datasets. Additionally, RF provides rankings of feature importance, which enables feature selection and effectiveness analysis [43,44]. 

##### K-Nearest Neighbors

K-nearest neighbors (KNN) is a non-parametric machine learning algorithm for classification or regression. It is a typical algorithm of lazy learning since it does not involve a training process while simply storing the training set samples, and other operations are performed in the stage of prediction. During the prediction phase, KNN requires traversing all the samples in the training set, which leads to a heavy computational workload. To improve the efficiency, we employ an acceleration strategy based on a K-dimensional (KD) tree. A KD tree is a binary tree data structure used to partition data into different regions in the feature space, aiming to reduce the computational cost of searching for nearest neighbors. When constructing a KD tree, we select the feature with the maximum distribution variance in the dataset and partition the dataset based on the median value of that feature’s distribution. This process is repeated until no further partitioning is possible, completing the construction of the KD tree. During the nearest neighbor search using the KD tree, the test sample is recursively searched based on the feature order selected during the construction of the KD tree. The algorithm continually updates the nearest training sample to the test sample based on feature distances. This process completes the nearest neighbor search [45,46]. The optimization effect of the KD tree-based acceleration strategy on the computational complexity of KNN will be demonstrated in Section 3.2.

##### Gaussian Naive Bayes

Naive Bayes (NB) is a generative classification method based on Bayes’ theorem and the assumption of conditional independence among features. It assumes that the features of a sample are independent of each other and learns the joint probability distribution of the class labels and the features. Then, it calculates the posterior probability of a new sample belonging to each class based on a certain feature distribution using Bayes’ formula. Finally, it determines the class label of the sample by maximum a posteriori (MAP) [47,48,49].

As a typical approach of NB, Gaussian naive Bayes (GNB) assumes that the features have continuous distributions, and the conditional probabilities of each feature dimension in the samples follow a Gaussian distribution, as shown in Equation (11).

(11) $$P(x_{i}|y_{c})=\frac{1}{\sqrt{2\pi \sigma_{ci}^{2}}}\exp\left(-\frac{{\left(x_{i}-\mu_{ci}\right)}^{2}}{2\sigma_{ci}^{2}}\right)$$

where *x_i_* denotes the *i*-th dimension feature and $\mu_{ci}$ and $\sigma_{ci}$ denote the mean and standard deviation of feature *x_i_* under the condition of category y to be c.

Based on the distribution assumption, Gaussian naive Bayes describes the differences between classes via the mean and variance of the features. The GNB model is simple, highly interpretable, suitable for incremental training, and efficient for large datasets.

##### Logistic Regression

Logistic regression (LR) uses the logistic function to map the output of linear regression to the probability space. The training goal for the LR model is to maximize the observed sample probability, so maximum likelihood estimation (MLE) is usually used to determine model parameters.

LR is an optimal meta-learner for stacking due to its combination of interpretability, efficiency, and robustness. As a linear model, LR provides a transparent understanding of feature weights and model outputs, facilitating the examination of decision-making processes. The statistical robustness of the LR model enables it to adapt to datasets with diverse sample distributions. Moreover, LR is capable of effectively addressing both linearly separable and nonlinearly separable problems, even in high-dimensional feature spaces. These attributes render logistic regression an appropriate candidate for the meta-learner role in stacking, ensuring a balance between performance and simplicity.

##### Summary

As outlined in Section 2.2.1, the selection and combination of base-learners should be informed by an understanding of the characteristics of the classification algorithms, with the objective of achieving complementary advantages. In general, RF and GNB exhibit lower variance, while KNN exhibits lower bias. Consequently, the meta-model exploits the respective strengths of these three algorithms to reduce bias and variance, thereby enhancing their generalization capacity. Furthermore, the training and inference efficiency of these three algorithms is superior to that of models such as SVM, which necessitate the resolution of complex quadratic programming problems during training. In conclusion, the configuration of the proposed algorithm for the selection of base-learners and meta-learners is set up based on a comprehensive consideration of performance and efficiency. This will be further validated in detail in Section 4.

## 3. Simulation Experiment Design

To validate the proposed signal modulation recognition method and evaluate its performance, we conduct a signal simulation based on the Simulink platform in MATLAB to obtain the data for the training and verification of the algorithm model. This section details the simulation configurations.

### 3.1. Classification Target and Data Set

To comprehensively evaluate the recognition performance of the proposed method for various signal modulation types, some typical wireless communication signal types were selected as the classification objects, including signals based on analog modulation and digital modulation. In the analog modulation signals, linear frequency modulation (LFM) is a typical linear modulation, while sine frequency modulation (Sine FM) is a nonlinear modulation, both of which are continuous waves. Linear frequency modulation within pulse (LFMP) is a common signal modulation for pulse radar. In digital modulation, binary phase shift keying (BPSK) is the most widely used modulation, which is characterized by high spectral efficiency and high data rate.

Furthermore, active jamming signal types, including amplitude modulation frequency sweep (AMFS) and noise modulation, were also employed as classification objects. Frequency sweep represents a typical form of active jamming, characterized by a wide frequency variation range that encompasses the frequency range of radar signals. It results in a periodic and significant disruption to the radar signal. A noise modulation signal is employed in a blanket jamming technique, wherein Gaussian noise is used for modulation. Noise frequency modulation (NFM) and noise amplitude modulation (NAM) are the typical implementations of noise modulation.

Table 1 provides the details of the modulation types mentioned above.

In the experiment, the aforementioned types of signal data were obtained through the process of simulation synthesis using Simulink. The sampling rate was set to 100 MHz. Each of the aforementioned types comprised a total of 1000 signal samples within the dataset. The length of a signal sample was 2000. Overall, 80% of the samples in the dataset were assigned to the training set, while the remaining 20% were assigned to the validation set. The parameters of the signal are presented in Table 2. 

In Table 2, the value given by the range means that the parameter value is obtained by random uniform sampling within the value range. The modulation frequency of LFMP is its intra-pulse LFM signal frequency and the duty ratio is determined by the period of its intra-pulse LFM signal and the pulse width. Sawtooth FM, abbreviated as Saw FM, denotes the multiple periodic continuous LFM signal. For NAM and NFM, the noise for modulation is processed by the filter and has a specific bandwidth defined by the modulation bandwidth. For Sine AMFS, its signal amplitude is modulated by a sine wave, with its frequency being the same as the modulation frequency.

Due to the use of cross-validation during the training of the meta-learner in the stacking algorithm, it is necessary to ensure a class balance between the training sets of the base-learner and meta-learner. Therefore, we set the proportion of each class signal in the sub-training set and sub-validation set obtained through cross-validation to be balanced and consistent.

### 3.2. Complexity Analysis

We conducted an analysis of the computational complexity of the proposed classification algorithm from two perspectives: time complexity and space complexity. This analysis covers the overall algorithm as well as the complexity of the base-learner and meta-learner during both training and prediction phases. The results of this analysis are presented in Table 3.

RF has a higher computational complexity during the training phase but a relatively low computational complexity during the prediction phase. The computational complexities of KNN during both training and prediction phases are directly related to the number of training samples, whereas for other algorithms, the relationship between computational complexity and the number of training samples only exists during the training phase. During the training phase, KNN does not involve significant computation but rather stores the training data. However, during the prediction phase, KNN needs to calculate the distances between the test sample and all training samples. Therefore, when dealing with large datasets or high-dimensional features, the computational complexity of KNN is high. By introducing a KD tree, it is allowed to reduce the searching complexity during the prediction phase from O(k·n) to O(k·log⁡n). GNB has a lower computational complexity during the training phase since it involves the calculation of prior probabilities and conditional probabilities for each class. During the prediction phase, it computes the posterior probabilities to each class for one sample, resulting in relatively low computational complexity. As the meta-learner of stacking, LR has a higher computational complexity during the training phase, as it requires iterative calculation for the optimization algorithm. During the prediction phase, its computational complexity is relatively low, as it only involves linear combinations of features and weights, as well as the calculation of activation functions.

In summary, GNB and LR have the highest computational efficiency, followed by KNN, while RF has the highest computational complexity during training. For the proposed stacking algorithm, the computational complexity is influenced by the base-learner, meta-learner, and the number of folds in cross-validation. Table 3 demonstrates that the number of folds in cross-validation has a substantial impact on the complexity. Hence, it is crucial to determine the value of *K* to strike a balance between algorithm prediction performance and computational efficiency.

### 3.3. Hyperparameter Optimization

#### 3.3.1. Optimization Target

This section discusses the hyperparameter optimization of the proposed algorithm, which includes optimizing the hyperparameters for the stacking algorithm model and its sub-models (base-learners and meta-learner). It involves first optimizing the hyperparameter configuration for each base-learner and then optimizing the configuration of the stacking model. It should be noted that the optimization of the meta-learner should be performed after the optimization of base-learners.

For the RF model, it is important to consider the number of decision trees, the criterion, and the maximum number of features. The number of decision trees has a significant impact on the overall scale of the model, which in turn affects its performance. Insufficient decision trees can lead to underfitting, while an excess can cause over-fitting and significantly increase computational complexity. The criterion is employed to assess the efficacy of the decision-tree splitting behavior. It is typically quantified using either the Gini impurity or the Shannon information entropy. The maximum number of features defines the upper limit for the number of features to be considered when identifying the optimal split for a decision tree. It is frequently set based on a function of the total number of features, such as the square root or logarithm of the total number of features.

For the KNN model, a larger value of K (number of neighbors) helps reduce the influence of noise but may result in blurred boundaries between classes [50]. Therefore, the *K* value should be determined based on the dataset. KNN often employs the Minkowski metric as the distance measure, which is defined as Equation (12).

(12) $$D(i,j)={\left[\sum_{k=1}^{K}{\left|x_{k}(i)-y_{k}(j)\right|}^{p}\right]}^{\frac{1}{p}}$$

where $x_{k}(i)$ and $y_{k}(j)$ denote the *i*-th and *j*-th elements of feature vectors x and y in the *k*-th dimension, respectively. *K* denotes the number of feature dimensions, and *p* denotes the order of the Minkowski metric. It is typical to set p as 1 or 2, which corresponds to the Manhattan distance or Euclidean distance, respectively. Furthermore, the utilization of distance weighting assigns more influence to neighbors that are closer in distance, compared to neighbors that are farther away.

For the GNB model, it provides features with the assumptions of conditional independence and the Gaussian distribution of priori. Thus, it is essential to specify the occurrence probabilities of each class in the dataset as the prior probabilities.

For the LR model, it is necessary to specify the norm of the penalty, regularization strength, and solver. The penalty is employed to control the complexity and generalization error of the model, reducing its sensitivity to noise in the training data and preventing over-fitting. The solver parameter is used to determine the choice of optimization algorithm.

#### 3.3.2. Optimization Method

Traditional hyperparameter optimization methods include grid search, stochastic grid search, halving grid search, and genetic algorithm. What these methods have in common is to validate all grid points in a large parameter space and then return the optimal loss function value. Although strategies such as stochastic and halving can shorten training costs and accommodate large data and large parameter spaces, these methods still fail to achieve win–win results in efficiency and accuracy. In order to search faster and find the hyperparameters with the best generalization ability, we adopt the Bayesian optimization (BO) method to automatically optimize the hyperparameters of the proposed model.

Bayesian optimization approximates the objective function by constructing an intelligent probabilistic model. It focuses on promising regions and gradually narrows down the search space, making the search for the optimal solution in the hyperparameter space more effective. It cleverly combines an exploratory search with the utilization of historical information, avoiding enumeration and significantly improving search efficiency. The process of Bayesian optimization can be summarized as modeling and optimization. In the optimization phase, the probabilistic model is updated based on the outputs obtained from the objective function for different inputs, and it serves as the posterior probability distribution, guiding the selection of the next sampling. This selection process aims to utilize the previously observed optimal values while also considering unexplored regions in the global parameter space. The selection strategy is typically defined by an acquisition function. Through the iterative process of modeling and optimization, Bayesian optimization gradually converges to the global optimal solution while minimizing the number of samples required from the objective function [51,52].

Algorithm 3 presents the process of Bayesian optimization for the hyperparameters of the proposed algorithm. By performing a Bayesian optimization process on each algorithm model, the hyperparameters required for that model are obtained. It is important to emphasize that each machine learning algorithm has its unique set of hyperparameters, and the quantities of these parameters differ. Therefore, the dimensionality of the vector *x* in Algorithm 3 adjusts according to the specific algorithm in consideration.
**Algorithm 3.** Bayesian hyperparameter optimization algorithm for the classification algorithm sub-model of the proposed method**Input:** Objective function f(x), search space χ of hyperparameter, sampling budget *n*, initial point of hyperparameter $x_{0}$, probability model *P*, acquisition function a(x)**Output:** Optimal hyperparameter set x* (multi-dimensional vector)**Define:** (1) Objective function f(x) is set to exhibit metric accuracy, as defined by Equation (13) in Section 3.4; (2) Probability model *P* is set to be Gaussian process model; (3) Acquisition function a(x) is set to be expected improvement (EI) function.1**Initialization**: $t=0,X_{t}=\{x_{0}\},Y_{t}=\{f(x_{0})\}$2**while** (t<n) **do**3  Obtain ($X_{t},\;\;Y_{t}$) by *P*4  Calculate the next sampling point $x_{t+1}={argmax}_{x\in X}\;a(x)$5  Evaluate the objective function $y_{t+1}=f(x_{t+1})$6  Update: $X_{t+1}=X_{t}\cup \{x_{t+1}\}$7  Update: $X_{t+1}=X_{t}\cup \{x_{t+1}\}$8  Update iteration step t=t+19**end while**10Output $x*={argmax}_{x\in X}\;f(x)$ as the optimal hyperparameter set x*

The hyperparameters of each model obtained by Bayesian optimization are shown in Table 4. It should be noted that the prior probability of the class required by the GNB model is determined by the dataset, so BO is not involved.

### 3.4. Evaluation Metrics

The results of a classification task are commonly represented using a confusion matrix, as illustrated in Table 5.

For the radar signal type classification task, the corresponding explanation of each case in Table 5 is as follows:

(1) True positive (TP): current signal type is correctly identified;
(2) True negative (TN): other signal types are correctly identified;
(3) False negative (FN): current signal type is misidentified as other signal type;
(4) False positive (FP): other signal types are misidentified as current signal type.

Based on the above, the overall performance of the proposed method is evaluated using four metrics: accuracy, precision, recall, and F1_score. These metrics are defined as shown in Equations (13)–(16), respectively. The higher the value of the metrics, the better the classification performance of the algorithm.

(13) $$\mathrm{Accuracy}=\frac{TP+TN}{TP+TN+FP+FN}$$

(14) $$\mathrm{Precision}=\frac{TP}{TP+FP}$$

(15) $$\mathrm{Recall}=\frac{TP}{TP+FN}$$

(16) $$F1\_\mathrm{score}=\frac{2\cdot Precision\cdot Recall}{Precision+Recall}$$

Accuracy provides an overall measure of prediction correctness, although it can be affected by class imbalance. Precision and recall focus on the model’s performance on positive samples and help evaluate specific prediction abilities. Precision measures the proportion of correctly predicted positive samples out of all samples predicted as positive, while recall quantifies the proportion of correctly predicted positive samples out of all actual positive samples. The F1-score combines precision and recall into a single metric and is suitable for evaluating the balance between both, allowing us to assess the trade-off between correctly identifying positive samples and minimizing false positives.

Furthermore, receiver operating characteristic (ROC) curves are used to analyze the recognition accuracy of the proposed method for each signal type. The classification algorithm model outputs confidence in the classes to which a sample might belong, and ROC curves are created by plotting the fraction of true positives out of the positives (true positive rate, TPR) vs. the fraction of false positives out of the negatives (false positive rate, FPR) at various threshold settings. TPR and FPR are respectively defined by Equations (17) and (18). The ordinate of the ROC curve is TPR, and a higher index represents a higher accuracy of recognition. The abscissa of the ROC curve is FPR, and a lower index represents a lower misrecognition rate. The area under the ROC curve is defined as the area under the curve (AUC). The closer the ROC curve is to the upper left corner, the larger the AUC and the higher the recognition accuracy.

(17) $$\mathrm{TPR}=\frac{TP}{TP+FN}$$

(18) $$\mathrm{FPR}=\frac{FP}{TN+FP}$$

## 4. Results and Analysis

### 4.1. Validity Analysis of Features

#### 4.1.1. Feature Importance Analysis

The feature importance analysis is performed to obtain the contribution of each feature to the classification model. To capture the nonlinear relationships and complex interactions between features, we employed the method of Gini impurity (GI) and permutation importance (PI) to evaluate feature importance.

The GI-based method is designed based on the RF model, and it evaluates the feature importance by measuring the decrease in Gini impurity when a feature tree node is split in a decision tree. Gini impurity denotes the probability of misclassifying a random sampling from a dataset, and the Gini impurity $I_{G}\left(D\right)$ at feature node D in a decision tree is defined by Equation (19).

(19) $$I_{G}(D)=\sum_{i=1}^{C}p(i)\left[1-p(i)\right]$$

where C denotes the total number of classes, and p(i) denotes the probability of the current sample belonging to the *i*-th class. A larger feature importance of GI indicates that the model’s classification performance would be significantly impacted if that feature was missing, implying that the feature is more important. The GI-based method is suitable for features with distinct boundaries, as it can accurately measure their contribution to the classification results.

The PI-based method evaluates the feature importance by randomly permuting the feature and observing the classification accuracy variation. It quantifies the importance of a feature based on the extent to which the model accuracy decreases when the feature is randomly permuted. The PI-based method is useful for capturing the impact of features with complex interactions or nonlinear relationships on the classification results. In the experiment, the random feature permutation was performed for 100 times to obtain an average value of the importance score.

**Figure 4:**
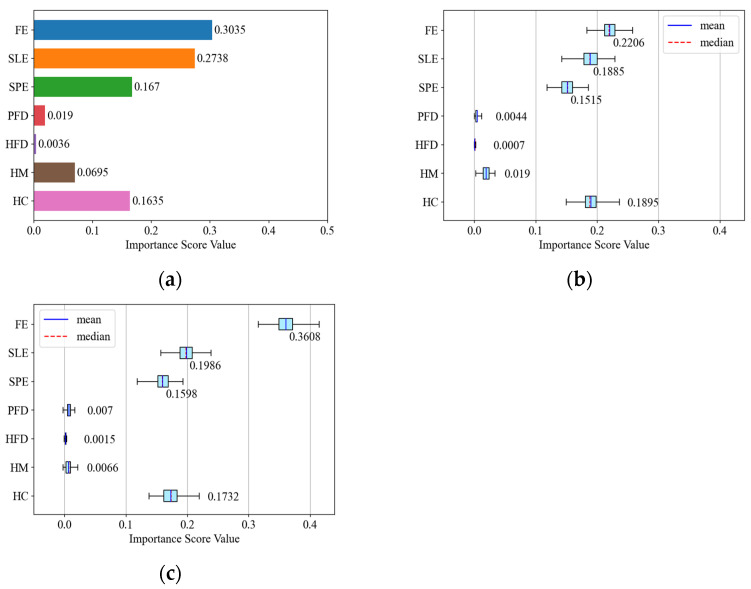
Statistics of feature importance scores: (**a**) importance score of the GI-based method; (**b**) importance score of the PI-based method (RF model); (**c**) importance score of the PI-based method (stacking model).

As the results of the GI-based method are computed based on the training set, they may not accurately reflect the generalization ability of the feature. Therefore, we assigned the dataset at an SNR of −10 dB to the training set and validation set. The GI-based method was applied to the training set, and the PI-based method was applied to the validation set. Figure 4 shows the importance statistics results based on the aforementioned dataset assignment. Figure 4a shows the results of the GI-based method. Figure 4b shows the results of the PI-based method. Figure 4c shows the results of the proposed stacking model.

From Figure 4, it can be seen that the feature importance scores obtained by the RF model and the stacking model are distinctly ranked through two different methods. The four most important features are FE, SLE, SPE, and HC. By comparing Figure 4b,c, it can be concluded that the relative trends of the feature importance of the RF model and the stacking model based on the method of PI are consistent with each other. And FE has the highest importance among the three results.

It is important to note that the feature importance metric is typically employed to assess the relative importance trend of each feature. The resulting values are contingent upon the specific dataset, which may exhibit certain distribution discrepancies. It is essential to recognize that a low importance score value does not necessarily imply that the feature is inconsequential for classification purposes.

#### 4.1.2. Impact of SNR on Feature Performance

**Figure 5:**
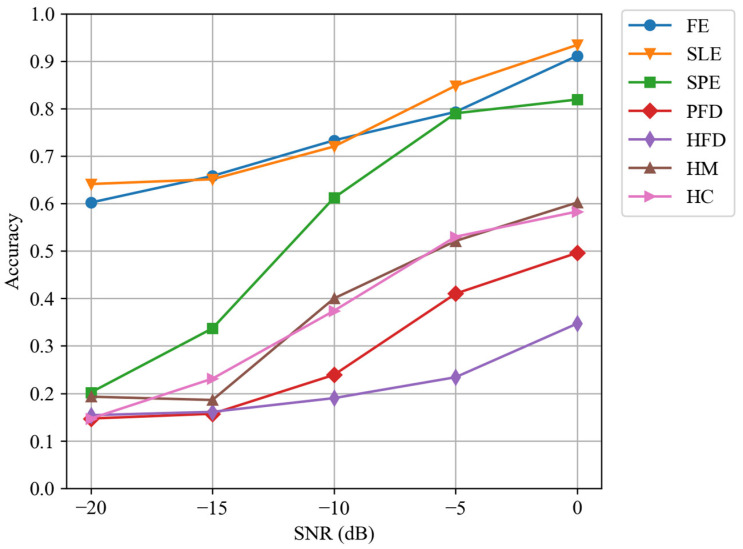
Impact of the SNR on the classification performance of features.

This section analyzes the impact of varied SNRs on the classification performance of each feature. Figure 5 illustrates the changes in classification accuracy using each feature by the proposed stacking method at SNRs of 0, −5, −10, −15, and −20 dB.

Figure 5 illustrates that the classification performance of various features is influenced differently by the SNR. The features FE and SLE consistently demonstrate the highest accuracy, significantly outperforming other features, regardless of the SNR level. On the other hand, the accuracy of SPE shows the greatest improvement with the increasing SNR, jumping from about 0.2 at an SNR of −20 dB to about 0.8 at an SNR of 0 dB, highlighting the SPE’s heightened sensitivity to SNR. The accuracies of HM and HC are quite close, and both show a significant improvement with the increase in SNR. Overall, the HFD exhibits the lowest accuracy among all features.

### 4.2. Performance Verification Experiment

In this section, two sets of ablation experiments are performed to evaluate the configuration effectiveness of features and the classification algorithm. Both experiments are performed with an SNR of −10 dB.

#### 4.2.1. Performance Verification of Features

The first experiment is based on the proposed classification algorithm of the improved stacking model. The aim is to validate the effectiveness of the feature combination. The results are presented in Table 6.

Table 6 demonstrates that an increase in the number of features utilized for classification leads to a corresponding improvement in classification performance, suggesting that the incorporation of each feature is justified and beneficial. Furthermore, the experiment also evaluated the classification performance of the proposed algorithm model using the signal feature from references [10,11]. The corresponding results show that their classification performances are inferior to those of the features proposed in this paper, which implies that the features employed in those two studies are not well suited for the signal classification tasks under the specific SNR conditions addressed in this paper.

#### 4.2.2. Performance Verification of Algorithm

The second experiment is to verify the performance of the proposed classification algorithm of the improved stacking model and its sub-learners. Other typical machine learning classification algorithms are also listed for reference. Furthermore, the effectiveness of the proposed meta-feature enhancement was also verified. Table 7 presents the experiment results. The metric of time cost represents the time required by the algorithm to process one sample.

Table 7 reveals that RF exhibits the highest performance within the non-ensemble learning algorithms, with GNB coming in second and DT being the least effective. In terms of inference speed, LR is the quickest, followed by DT. KNN is the slowest. For the concerned signal classification task, the GNB offers the most cost-effective solution. The utilization of the stacking-based algorithm results in a discernible improvement in classification performance, even when employing one classification model as the base learner. Furthermore, the application of meta-feature enhancement leads to a further performance enhancement, with the four metrics achieving a score of 0.988. Since the meta-feature enhancement is intended for the meta-learner and LR is already rapid in its inference speed, there is no significant additional time cost. When compared to other ensemble learning algorithms such as gradient boosting decision trees (GBDT) and adaptive boosting (AdaBoost), the proposed stacking-based algorithm demonstrates a distinct performance advantage.

### 4.3. Adaptability Testing for Application Scenarios

#### 4.3.1. Impact of SNR on Classification Performance

This section presents the experiment to evaluate the performance of the proposed classification method at various SNRs. The proposed classification algorithm model was trained and verified separately at each SNR. The results are presented in Table 8.

From Table 8, it can be seen that the proposed method achieves excellent classification performance at SNRs of 0 dB, −5 dB, and −10 dB. Specifically, it achieves 100% accuracy in identifying all signal types at SNRs of 0 dB and −5 dB. Furthermore, the performance of the proposed classification method shows a decrease of about 20% when comparing an SNR of −15 dB with one of −10 dB and a decrease of about 15% when comparing an SNR of −20 dB with one of −15 dB. In summary, the proposed classification method shows ideal performance even in practical application scenarios with a low SNR.

**Figure 6:**
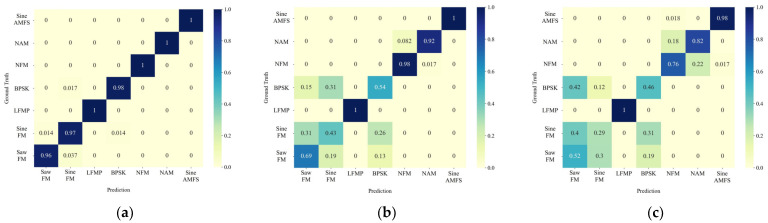
Confusion matrices of classification results of the proposed method at different SNRs: (**a**) confusion matrix at an SNR of −10 dB; (**b**) confusion matrix at an SNR of −15 dB; (**c**) confusion matrix at an SNR of −20 dB.

**Figure 7:**
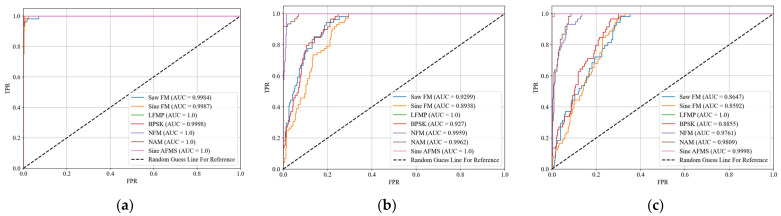
ROC curves of the classification results of the proposed method at different SNRs: (**a**) ROC curve at an SNR of −10 dB; (**b**) ROC curve at an SNR of −15 dB; (**c**) ROC curve at an SNR of −20 dB.

Moreover, we analyze the trend of the identification accuracy of the proposed method for each signal type as the SNR varies, utilizing the confusion matrix depicted in Figure 6 and the ROC curve presented in Figure 7.

Figure 6 illustrates that the proposed method achieves excellent classification performance at an SNR of −10 dB. LFMP and three types of active jamming signals can be completely distinguished. As the SNR decreases to −15 dB and −20 dB, the probability of confusion increases, and confusion also arises among the signals of NFM, NAM, and Sine AMFS. Furthermore, regardless of the SNR, the proposed method does not exhibit any confusion between the radar signals and active jamming signals.

From Figure 7, it can be seen that there is a noticeable decrease in the AUC for the signals of Saw FM, Sine FM, and BPSK as the SNR decreases, followed by NFM and NAM. However, the AUCs for LFMP and Sine AMFS remain consistently high. Importantly, even after the decline, the ROC curves for all signal types remain significantly higher than the dotted line, which represents the random guess line.

#### 4.3.2. Performance Evaluation of Few-Shot Learning

**Figure 8:**
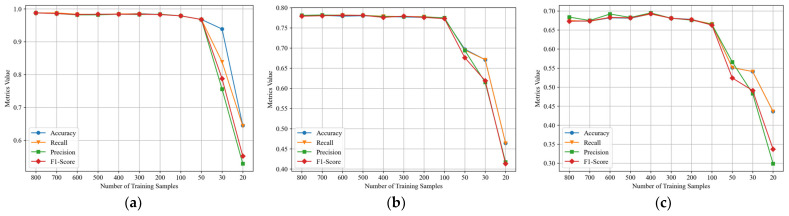
Trends of the proposed method’s classification performance with varied sizes of training set at different SNRs: (**a**) trend of classification performance at an SNR of −10 dB; (**b**) trend of classification performance at an SNR of −15 dB; (**c**) trend of classification performance at an SNR of −20 dB.

In the previous sections, the experiments were performed with 800 training samples and 200 validation samples for each signal type. In this section, we keep validation samples unchanged while reducing the number of training samples, and then the proposed algorithm model is trained and evaluated. This could be regarded as the performance evaluation of the scenario of few-shot learning. Three sets of experiments are performed at SNRs of −10, −15, and −20 dB, and the results are shown in Figure 8.

From Figure 8, it is evident that at the three different SNRs, with negligible data fluctuations caused by small variances within an acceptable range, the proposed method can be considered to maintain consistent performance when the size of training set exceeds 100. However, a noticeable decrease in performance is observed when the size of the training set is reduced to 50 or even smaller. In other words, for the concerned task scenario and target to be recognized in this research, the proposed signal classification model only requires 100 training samples to learn sufficient information and achieve convergence in model training. This significantly reduces the pressure and cost of dataset construction compared to the common practice of deep neural network models, which often require datasets with more than ten thousand samples. The proposed method maintains competitive performance even in the scenario of few-shot learning, enabling the training of an effective classification model for signal modulation types with only a small amount of real data.

**Figure 9:**
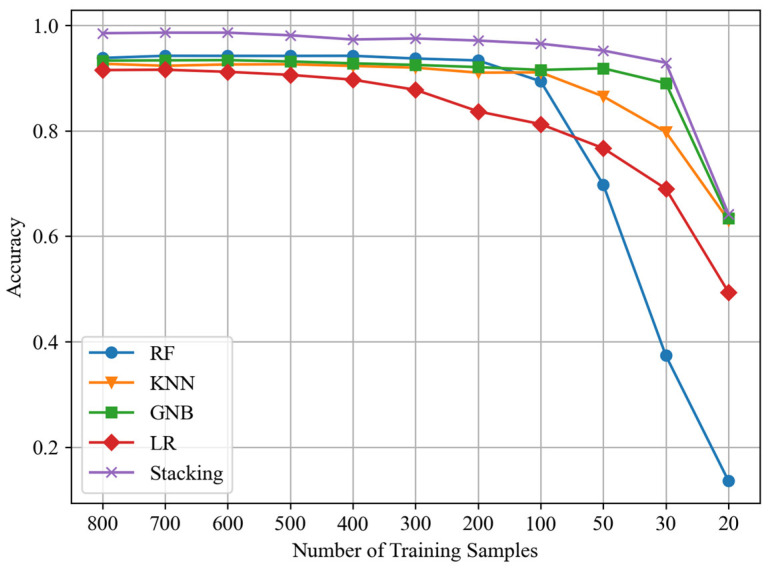
Trends of each sub-learner algorithm model’s classification performance with different sizes of the training set at an SNR of −10 dB.

In order to explore the performance support of the proposed method in the scenario of few-shot learning, we conducted performance tests on individual sub-learners within the stacking framework at an SNR of −10 dB. This helps to evaluate their contributions to the proposed stacking model. The dataset configuration remained consistent with previous setups, and results were evaluated based on accuracy, as shown in Figure 9.

From Figure 9, it can be observed that the RF and LR models are significantly affected by a reduction in the number of training samples. However, their accuracy variation trends are different. Specifically, the RF model shows a noticeable performance decline only after the training sample number decreases to 100, and its decline magnitude is the largest among all models. On the other hand, the LR model’s performance gradually decreases with fewer training samples, exhibiting a relatively gentle decline. Among all models, the performance of KNN and GNB is least affected by the decrease in training samples, and GNB’s performance only significantly drops when the number of training samples is below 30. KNN and GNB make the primary contributions to the performance of the proposed stacking model in the scenario of few-shot learning.

### 4.4. Contrast Experiment

The performance comparison of the proposed method with other signal modulation recognition methods is provided in this section to evaluate the performance differences between the proposed method and other methods in the scenarios of a low SNR and few-shot learning. The involved methods include MAPNet from reference [14], MSCANet from reference [18], DNCNet from reference [19], and CLDN from reference [20]. These are different types of neural network models, including the general CNN, the CNN with an attention mechanism, and a composite model combining the CNN, DNN, and LSTM. In terms of structural design, they incorporate distinct functional modules like a noise estimation module and a denoising module. Regarding the input data format, some methods process one-dimensional time-domain signals, while others process two-dimensional time-frequency distributions of the signals.

**Figure 10:**
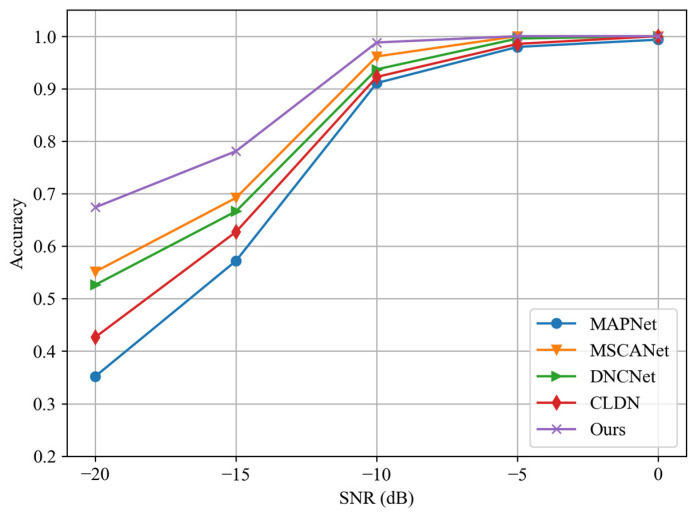
Classification performance comparison between the proposed method and other methods at different SNRs.

Figure 10 shows the classification accuracy of the aforementioned methods at different SNRs. At higher SNRs, the signal features are more prominently distinguishable, allowing each method to identify the signal modulation type with high accuracy. At this time, they do not exhibit obvious performance divergence. For example, when the SNR is −5 dB, the classification accuracy of all methods is above 95%. As the SNR gradually decreases, the classification accuracies of all methods decrease, and the performance differences between various methods are amplified. When the SNR is −15 dB, the classification accuracy of our method is 78.1%, while the lowest-performing MAPNet achieves only 57.21%, and when the SNR is −20 dB, the accuracy of MAPNet is only 35.21%.

Among the compared methods, MSCANet and DNCNet, which are designed with noise-resistant models, exhibit a certain accuracy advantage over the other two methods. By leveraging a combination of noise-resistant features to characterize signals and employing the ensemble learning approach, our method achieves optimal performance at different SNRs. Moreover, as the SNR decreases, the performance advantage of our method over other methods continues to expand, highlighting the robustness of our approach for low-SNR task scenarios.

Furthermore, following the approach outlined, we conduct an in-depth comparison of the aforementioned methods in terms of sample feature representation and classification capabilities. For signal classification methods based on deep neural network models, they are generally regarded as black boxes due to their end-to-end execution of feature extraction and category prediction using input signal samples. Let us consider the fully connected layer of the neural network model as the classifier, while the remaining parts of the model can be seen as the feature extractor. The feature extractor extracts latent information from the input raw data to characterize signals and provides the extracted features to the classifier in the form of a vector with a specific dimension. The fully connected layer typically has a high dimension, consistent with the dimension of the feature vector it receives.

The nonlinear dimensionality reduction method of t-distributed stochastic neighbor embedding (t-SNE) was employed to reduce the high-dimensional feature vectors involved in the aforementioned methods. This included the inexplicable high-dimensional feature vectors in deep neural network models and various features used in this paper. The feature vectors were reduced to the format of a two-dimensional vector and then visualized. The objective of this processing was to analyze the discriminative ability of these features for different modulation types in signal samples. Furthermore, through Monte Carlo simulations, we obtained the classifier’s decision boundaries in the two-dimensional vector space.

**Figure 11:**
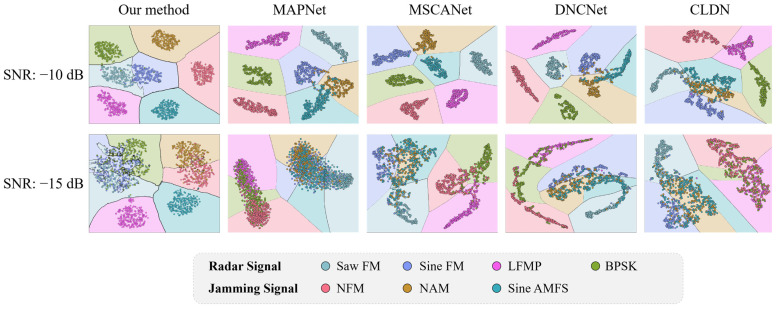
Dimensionality reduction visualization of feature vectors and comparison of classification decision boundaries of different classification methods.

Figure 11 presents a dimensionality reduction visualization of the signal features extracted by the aforementioned methods, along with their classification decision boundaries. Each point in the figure represents the distribution of a single sample, while the colored areas indicate the decision regions predicted by the classification algorithms. The distribution location and clustering degree of the sample points reflect the feature extraction capabilities of the classification methods for various modulation types of signal samples. The degree to which the decision boundary matches the scatter range of the signal sample points represents the performance of the classification algorithm in distinguishing different types of modulation signal samples. In order to provide a more accurate representation of the performance differences, we have included the results of contrast experiments at SNRs of −10 dB and −15 dB.

Figure 11 illustrates the effectiveness of various signal sample clustering methods at an SNR of −10 dB. It can be observed that all methods achieved effective clustering of various signal sample types, with minimal sample classification confusion. Additionally, the decision boundaries exhibited high consistency with the locations of the sample clusters. However, a noticeable decline in clustering ability was observed across all methods to varying degrees when the SNR dropped to −15 dB.

Specifically, our method exhibited some confusion between BPSK and Sine FM signals, as well as minor confusion between NAM and NFM signals. Nevertheless, the decision boundaries also underwent corresponding localized adjustments to optimally separate the samples that exhibited clustering confusion, such as the decision boundary for Sine FM. This behavior reflects the powerful nonlinear classification capability of the proposed stacking-based classification algorithm. In contrast, for the other methods, the decrease in the SNR led to more severe clustering confusion, and the classification decision boundaries based on fully connected layers struggled to effectively distinguish these confusions. Moreover, even with the decrease in the SNR, our method did not experience any clustering confusion or misclassification between radar signals and active jamming signals, demonstrating an advantage over the other approaches. In contrast, all four of the comparative methods exhibited such confusion. For instance, all four comparative methods suffered from clustering confusion and misclassification between Saw FM and NAM. Overall, our method exhibited the best performance in Figure 11, followed by the noise-resistant MSCANet and DNCNet, while MAPNet performed the worst.

**Figure 12:**
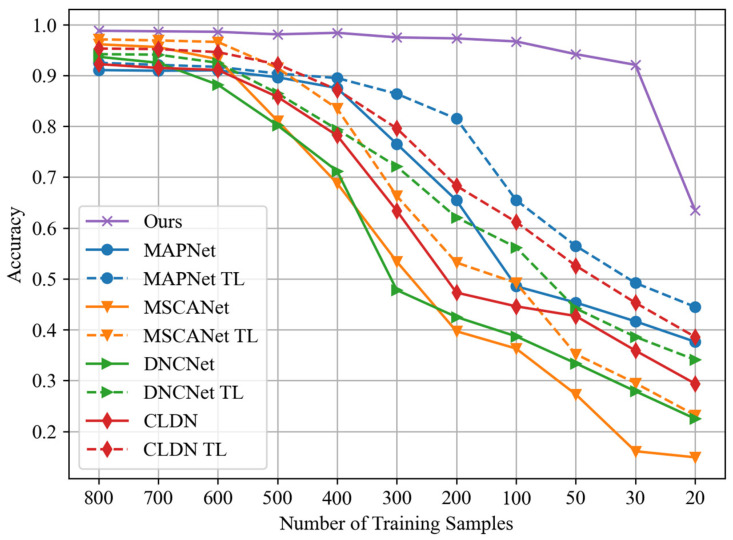
Classification performance variation trend of the proposed method and other methods with varying scales of the training set at an SNR of −10 dB.

Subsequently, the performances of the aforementioned methods are evaluated in the few-shot learning scenario at an SNR of −10 dB, with an experiment configuration similar to the experiments in Section 4.3.2. Specifically, for the neural network-based methods used for comparison, we first directly trained the neural network at an SNR of −10 dB and evaluated the performance. Subsequently, we applied the few-shot learning approach based on transfer learning (TL) to these methods. It involved completing the model training at an SNR of 0 dB first, then applying fine-tuning for the pre-trained models at an SNR of −10 dB, and finally conducting the performance evaluation. This enables the observation of variations in classification accuracy. The results are presented in Figure 12.

Figure 12 illustrates that as the scale of the training set decreases, the classification accuracy of the four neural network-based classification methods declines at a noticeably faster rate than that of the proposed ensemble learning-based method. This phenomenon is expected, as neural network models rely on data-driven updates of weight parameters, which directly impact their feature extraction and classification capabilities. Upon reducing the training sample size to 100, the accuracy of our method begins to decline significantly, while that of each neural network-based method has already dropped by more than half compared to when the sample size was sufficient, falling below 0.5. Furthermore, it can be observed that the relative performance trends of the four methods stabilize after the number of samples decreases below 200. At this stage, MSCANet has the lowest accuracy, while MAPNet achieves the highest accuracy. This relative performance trend is in direct opposition to that observed in Figure 10. We attribute this phenomenon to the fact that MSCANet, which exhibited the best performance among the four methods at the general training setup, has the largest network structure scale. Consequently, the convergence of its network weight parameter updates relies on a larger number of samples and more training iterations, which contrasts with the behavior of MAPNet. The insufficient number of training samples prevents the neural network models from attaining a convergent state during training. In this context, the smaller and simpler models are more adept at approximating their converged training performance. Furthermore, by adopting transfer learning, the accuracy of the neural network-based method increases compared to that when transfer learning was not used. This suggests that transfer learning enhanced the performance of the neural networks in few-shot learning scenarios to some extent and mitigated the trend of performance decline as the number of training samples decreased. Nevertheless, despite the incorporation of transfer learning, the performances of these neural network-based methods in few-shot learning scenarios are still limited and below the performance of the proposed method.

Furthermore, we provide the performance evaluation results of the aforementioned methods based on the benchmark of RadioML2016.10a, which is a commonly used open dataset for signal modulation recognition. The number ratio between its training set and validation set is consistent with that of our data set.

**Figure 13:**
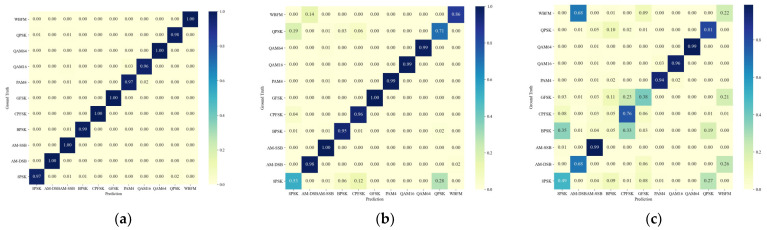
Confusion matrices of the classification results of the proposed method based on the dataset of RadioML2016.10a at different SNRs: (**a**) confusion matrix at an SNR of 6 dB; (**b**) confusion matrix at an SNR of 0 dB; (**c**) confusion matrix at an SNR of −6 dB.

Figure 13 shows the confusion matrices based on the proposed method’s classification results for the dataset RadioML2016.10a at SNRs of 6 dB, 0 dB, and −6 dB. Table 9 provides the performance evaluation results of the proposed method on the dataset RadioML2016.10a using the four metrics.

**Figure 14:**
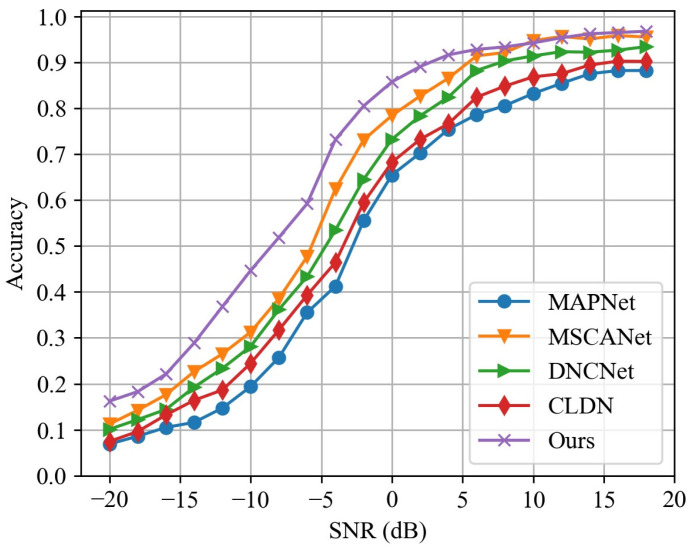
Classification performance comparison between the proposed method and other methods based on the dataset of RadioML2016.10a at different SNRs.

**Figure 15:**
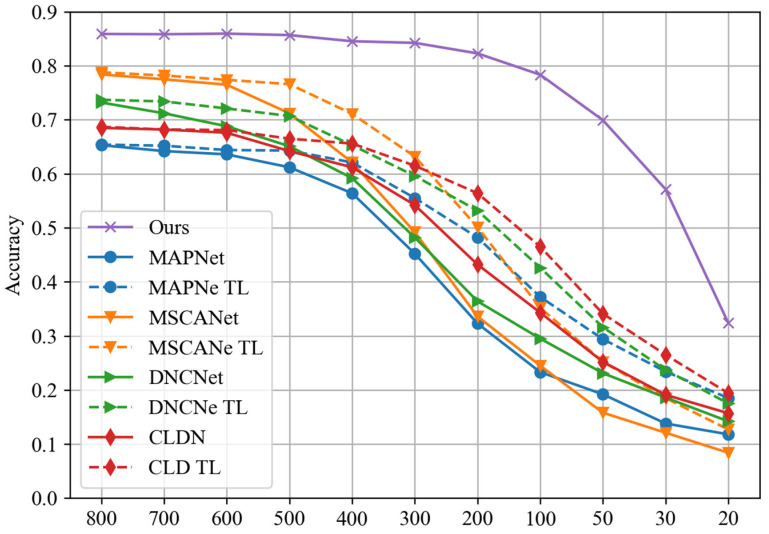
Classification performance variation trend of the proposed method and other methods with varying scales of the training set for RadioML2016.10a at an SNR of 0 dB.

We also provide the evaluation results of the compared methods’ performance at the scenarios of low SNR and few-shot learning based on the dataset of RadioML2016.10a. These experiment configurations are consistent with those of Figure 10 and Figure 11, respectively. Figure 14 shows the classification accuracy of the compared methods for dataset RadioML2016.10a at different SNRs. Figure 15 show the compared methods’ classification accuracy variations with a number decrease in the training set.

The performance evaluation results derived from the public dataset indicate that the performance trends of the proposed method are mainly consistent with the results based on our dataset. In conclusion, the above experiments have validated the advantages of the proposed method in terms of feature extraction and classification capabilities relative to the other approaches. Through performance comparisons and analyses, we have demonstrated the superiority of the proposed method over the other methods in the scenarios of a low SNR and few-shot learning. The proposed method is a classification algorithm of machine learning that exhibits strong robustness to noise and lower reliance on sample size, making it more adaptable compared to other methods for signal modulation recognition tasks in real-world radar countermeasure and anti-jamming scenarios.

## 5. Discussion

### 5.1. About Classification Performance

#### 5.1.1. Algorithm and Classification Performance

The essence of ensemble learning lies in managing the relationships among sub-models. Stacking utilizes a meta-learner to fuse the decision results of sub-models. Stacking’s decision fusion method is more accurate and reliable compared to traditional approaches such as voting, averaging, and weighted averaging. When the meta-learner is implemented by linear regression, it essentially solves the weighted sum of the decision results from all sub-models. The process of training the linear regression model involves finding the optimal weights. When the meta-learner is implemented by logistic regression, the decision results of the sub-models are transformed into probability values by a combination of linear operations and the sigmoid function. The optimal weights are then obtained through maximum likelihood estimation. This approach results in a more precise outcome.

Ensemble learning also addresses the issue of feature parameters. Bagging trains sub-models on bootstrap samples to reduce variance, which is suitable for high-variance base-learners. Boosting utilizes the output of the previous level as the input for the sub-models to reduce bias, so it is suitable for high-bias base-learners. As a structural improvement measure for the two-stage stacking model, meta-feature enhancement can be considered a form of feature identity mapping, similar to the residual connection structure in the DCNN model, allowing for the flow of original feature data across layers. The introduction of meta-feature enhancement aims to enable the meta-learner to simultaneously utilize the decisions of base-learners and the information from original features while avoiding biased guidance from potential common prediction biases of base-learners. The proposed stacking-based improved model combines the advantages of both bagging and boosting models, resulting in better classification performance.

#### 5.1.2. Feature and Classification Performance 

The majority of existing studies on radar signal modulation classification have focused on signal features based on a single feature domain. In contrast, the proposed method takes multiple types of features into account, including time-domain entropy, frequency-domain entropy, fractal theory-based self-similarity, and time-domain-based statistical properties. By considering these features, the classification algorithm gains a more comprehensive understanding of the signal characteristics. This enables the algorithm to extract more information even when the signal-to-noise ratio (SNR) is low, thereby leading to enhanced performance.

### 5.2. About Anti-Noise

This proposed method exhibits good performance in the signal modulation classification task under the condition of a low SNR. This is mainly achieved through feature engineering. To handle the noise, we select the features with strong noise resistance. From Figure 5, it is obvious that FE and SLE are the features that make the main contributions to anti-noise. Regarding FE, as mentioned in Section 2.1.1, it can be considered an improved version of sample entropy and approximate entropy. Its key improvement lies in utilizing an exponential fuzzy membership function as a measure of fuzziness. Compared to the previously used unit step function, the continuity of the exponential function results in a smoothly changing fuzzy entropy value with parameter variations. This enables FE to more accurately describe the prominence of signals with certain characteristics. Furthermore, this continuous variation in FE corresponds to the valuing characteristics of real-world physical quantity because the category boundaries of real signals described based on certain features are often continuous and fuzzy. The continuous values of fuzzy entropy can be regarded as a probability value, representing the confidence that the current signal belongs to a certain category. As a result, FE offers a greater amount of information to the classification algorithm, enhancing the likelihood of obtaining correct classification results. On the other hand, FE reduces the impact of data noise and outliers by averaging over sub-sequences. In summary, the unique computation method of FE provides it with excellent robustness against noise, as validated in Section 4.1.2. SLE achieves signal characteristic representation by calculating the entropy of the slope distribution of sub-sequences. As we know, the slope represents the global trend of the signal, so it is hard to destroy by the noise, trend of which (i.e., mean) is zero. In this process, the local impact of noise could be mitigated by the statistical method of slope within the sub-sequence interval. Moreover, the statistical analysis of interval slopes corresponding to multiple sub-sequences also helps to reduce the errors introduced by noise.

Recent studies have used time-frequency distribution images and CNN models for classifying radio and radar signals. These methods depend on clear signal components in time-frequency images and the strong noise resistance of the analysis methods. High SNR enhances signal intensity in these images, combining with CNNs’ feature extraction to achieve high accuracy. However, a low SNR concentrates energy on noise, depriving CNNs of sufficient features, leading to reduced accuracy. The two-dimensional time-frequency analysis may amplify noise’s disruptive effect on signals more than one-dimensional representations (time or frequency domains), as it allows local noise to obscure valuable information. On the other hand, despite noise’s local impact, it struggles to alter signals’ global trends. CNNs focusing on local features are vulnerable to noise’s bottom-up influence. In contrast, this paper’s feature extraction, based on signals’ global characteristics, offers noise resistance.

### 5.3. About Few-Shot Learning

As for the task of few-shot learning, we believe that traditional machine learning algorithms still have certain advantages compared to methods using deep neural networks, which are purely data-driven. In fact, the processes of machine learning algorithms include not only the algorithms themselves but also feature engineering. Feature engineering is achieved based on a priori information and expert knowledge, which are direct, effective, and interpretable. Therefore, feature engineering is efficient and controllable, compared to the deep learning methods that require finding potential features from a large amount of sample data. Thus, in cases where the number of features is much lower than the number of samples, its reliance on sample data is lower than that of deep learning methods.

On the other hand, the characteristics of the used sub-learner algorithm model also contribute to the adaptability of the proposed method in the few-shot learning scenario. GNB inherently carries a widely applicable prior assumption (i.e., the conditional independence assumption among features), which has been proven effective in multiple fields. GNB only requires estimating the mean and variance of features for each class, which makes it insensitive to noise and outliers. Thus, this also enhances its robustness in few-shot learning. As a non-parametric method, KNN does not require training, and the training set samples are only used as the reference for distance calculation, making KNN inherently suitable for few-shot learning. Furthermore, KNN does not make assumptions about sample data distribution, so it is not limited by the strict assumption of parametric models. This characteristic also enables KNN to adapt to small-scale training sets. Finally, KNN determines the category of a test sample by calculating its distance from each neighboring node (i.e., training sample) in the feature space. In other words, even with a small number of available samples, as long as each neighboring node has at least one sample, KNN can still make relatively reliable predictions.

## 6. Conclusions

This paper proposes an ensemble learning-based method for the signal modulation type identification of radar and jamming in the scenario of a low SNR and few-shot learning. The method contributes to algorithm design and feature engineering. It utilizes a stacking classification model with RF, KNN, and GNB algorithms as base-learners and a logistic regression algorithm as the meta-learner. The method proposes meta-feature enhancement to improve classification accuracy. Moreover, multiple features based on the time domain, frequency domain, and fractal domain serve as inputs for the algorithm, including fuzzy entropy, slope entropy, spectral entropy, fractal dimension, and Hjorth parameters, which provide a better upper limit of classification accuracy in comparison to single-domain features. The simulation experiments demonstrate that the proposed method achieves ideal accuracy for the classification task of seven signal types, including common radar modulation signals and active jamming signals, at different SNRs: 100% at −5 dB, 98.8% at −10 dB, 78.1% at −15 dB, and 67.4% at −20 dB. The expected performance can be achieved with only 100 training samples, and the inference time cost for one sample only takes 33 μs. The proposed method satisfies the requirements for recognition accuracy, running efficiency, a low SNR, and few-shot learning, making it available for real-world applications.

## Figures and Tables

**Table 1:** Classification objects: typical radar modulation signal types and active jamming signal types.

	Signal Modulation	Definition Formula	Description
Radar analog modulation	Linear frequency modulation (LFM)	$S\left(t\right)=cos\left[2\pi \left(f_{c}t+\frac{1}{2}kt^{2}\right)\right]$	$f_{c}$: Center frequency k: Modulation slope
	Sine frequency modulation (Sine FM)	$S\left(t\right)=cos\left[2\pi f_{c}t+m_{f}\int_{0}^{t}cos\left(2\pi f_{m}t\prime \right)dt\prime \right]$	$f_{c}$: Center frequency $m_{f}$: Modulation index $f_{m}$: Sine wave frequency
	Linear frequency modulation within pulse (LFMP)	$S(t)=rect(\frac{t}{T})\cdot cos\left[2\pi \left(f_{c}t+\frac{1}{2}kt^{2}\right)\right]$	rect(·): Rectangular wave function T: Pulse width
Radar digital modulation	Binary phase shift keying (BPSK)	$S(t)=cos(2\pi f_{c}t+\frac{2n\pi }{M}),n=0,1,2,\ldots ,M-1$	n: Phase state index M: Number of phase states S(t) is a BPSK signal when M=2.
Active jamming	Noise frequency modulation (NFM)	$J(t)=cos\left[2\pi \left(f_{c}t+m_{j}\int_{0}^{t}\;u_{n}(t^{\prime })dt^{\prime }\right)\right]$	$m_{j}$: Modulation index $u_{n}(t)$: Noise used for frequency modulation
	Noise amplitude modulation (NAM)	$J\left(t\right)=\left[U_{j}+U_{n}\left(t\right)\right]cos\left(2\pi f_{c}t\right)$	$U_{j}$: Carrier wave amplitude $U_{n}(t)$: Noise used for amplitude modulation
	Amplitude modulation frequency sweep (AMFS)	$J\left(t\right)=U_{j}\left(t\right)cos\left(2\pi f_{c}t+\theta \left(t\right)\right)$	$U_{j}\left(t\right)$: Amplitude modulation function θ(t): Sweep function

**Table 2:** Simulation parameter of each signal type.

Signal Type	Modulation Frequency (kHz)	Modulation Bandwidth (MHz)	SNR (dB)	Others
LFMP	[100, 200]	[10, 20]	0, −5, −10, −15, −20	Pulse width: 200 ns Duty ratio: [20%, 50%]
Saw FM	[100, 200]	[10, 20]	0, −5, −10, −15, −20	-
Sine FM	[100, 200]	[10, 20]	0, −5, −10, −15, −20	-
BPSK	-	-	0, −5, −10, −15, −20	Symbol width: [50, 90] ns
NAM	-	[10, 20]	0, −5, −10, −15, −20	-
NFM	-	[10, 20]	0, −5, −10, −15, −20	-
Sine AMFS	-	[10, 20]	0, −5, −10, −15, −20	-

**Table 3:** Computational complexity analysis of the introduced algorithms.

Algorithm	Time Complexity		Space Complexity
	Training Phase	Prediction Phase	
RF	O(n·log(n)·f·t)	O(d·t)	O(p·t)
KNN	O(f·n·log(n))	O(h·log(n))	O(n·f)
GNB	O(n·f·c)	O(c·f)	O(c·f)
LR	O(f·n)	O(f)	O(f)
Proposed algorithm	Training phase: O(K·(n·log(n)·f·t+f·n·log(n)+n·f·c)+f·n) Prediction phase: O(K·(d·t+h·log(n)+c·f)+f) Space complexity: O(K·(p·t+n·f+c·f)+f)		
Notes	Common	n: Number of training samples f: Number of features	
	RF	t: Number of trees p: Number of nodes in the tree d: Depth of the tree	
	KNN	h: Number of neighbors Acceleration strategy: KD tree	
	GNB	c: Number of classes to be classification	
	Stacking	K: Number of folds	

**Table 4:** Hyperparameters of models obtained by Bayesian optimization.

Algorithm Model	Hyperparameters	Value
RF	Number of decision trees	10
	Criterion	Gini
	Min samples of leaf	10
	Min samples of split	5
	Max number of features	sqrt
KNN	Number of neighbors	10
	Weights	distance
	Algorithm	KD tree
	*p* value	1
GNB	None	
LR	Norm of the penalty	L2
	Regularization strength	0.9
	Solver	LBFGS
	Max iteration	200
Stacking	Base-learner	KNN, RF, GNB
	Meta-learner	LR
	Fold of cross-validation	3
	Stack method	auto

**Table 5:** Confusion matrix for classification algorithm evaluation.

Confusion Matrix		Prediction	
		Positive	Negative
Ground truth	Positive	TP	FN
	Negative	FP	TN

**Table 6:** Comparison of classification performance based on single features and combinations of features.

Index	Feature	Accuracy	Recall	Precision	F1-Score
1	FE	0.732	0.733	0.745	0.732
2	SLE	0.722	0.720	0.717	0.718
3	SPE	0.612	0.612	0.592	0.598
4	PFD	0.239	0.239	0.217	0.207
5	HFD	0.190	0.190	0.191	0.170
6	HM	0.400	0.400	0.360	0.370
7	HC	0.374	0.373	0.391	0.367
8	FE + SLE	0.769	0.769	0.770	0.769
9	FE + SLE + SPE	0.911	0.911	0.914	0.910
10	FE + SLE + SPE + PFD	0.918	0.918	0.918	0.918
11	FE + SLE + SPE + PFD + HFD	0.921	0.921	0.921	0.921
12	FE + SLE + SPE + PFD + HFD + HM	0.966	0.964	0.965	0.965
13	FE + SLE + SPE + PFD + HFD + HM + HC	0.988	0.988	0.988	0.988
14	Features proposed in reference [10]	0.631	0.632	0.625	0.622
15	Features proposed in reference [11]	0.601	0.603	0.589	0.587

**Table 7:** Comparison of classification performance between single classification algorithms and ensemble classification algorithms based on stacking.

Index	Algorithm		Accuracy	Recall	Precision	F1-Score	Time Cost (us)
1	Selected algorithms without stacking	RF	0.938	0.939	0.938	0.939	6
2		KNN	0.927	0.927	0.928	0.927	22
3		GNB	0.933	0.934	0.934	0.934	0.6
4		LR	0.915	0.916	0.917	0.916	0.3
5	Other algorithms	DT	0.871	0.867	0.875	0.869	0.6
6		SVM	0.929	0.928	0.911	0.926	14
7		GBDT	0.932	0.933	0.934	0.932	11
8		AdaBoost	0.955	0.954	0.968	0.956	8
9	Stacking using meta-learner of LR and base-learners including:	RF	0.953	0.953	0.954	0.953	8
10		KNN	0.942	0.942	0.943	0.942	23
11		GNB	0.948	0.949	0.949	0.949	0.7
12		RF + KNN	0.963	0.963	0.965	0.964	32
13		RF + KNN + GNB	0.977	0.976	0.977	0.977	33
14		RF + KNN + GNB (with meta-feature enhancement)	0.988	0.988	0.988	0.988	33

**Table 8:** Classification performance comparison of the proposed method at different SNRs.

SNR (dB)	Accuracy	Recall	Precision	F1-Score
0	1	1	1	1
−5	1	1	1	1
−10	0.988	0.988	0.988	0.988
−15	0.781	0.781	0.781	0.779
−20	0.674	0.675	0.684	0.673

**Table 9:** Classification performance comparison of the proposed method and other methods based on the dataset of RadioML2016.10a at an SNR of 0 dB.

Method	Accuracy	Recall	Precision	F1-Score
MAPNet [14]	0.653	0.661	0.624	0.645
MSCANet [18]	0.784	0.782	0.778	0.779
DNCNet [19]	0.732	0.738	0.728	0.725
CLDN [20]	0.685	0.677	0.688	0.663
Ours	0.857	0.855	0.862	0.849

## Data Availability

The data are available from the corresponding author upon reasonable request.
